# METTL3-mediated NDUFB5 m6A modification promotes cell migration and mitochondrial respiration to promote the wound healing of diabetic foot ulcer

**DOI:** 10.1186/s12967-024-05463-6

**Published:** 2024-07-09

**Authors:** Tao Wang, Xu Li, Yue Tao, Xiaojun Wang, Limeng Li, Jianjun Liu

**Affiliations:** Department of Vascular Surgery, Qingpu Branch of Zhongshan Hospital, affiliated to Fudan University, 1158 East Park Road, Qingpu District, Shanghai, 201700 China

**Keywords:** Diabetic foot ulcer, NDUFB5, m6A, METTL3, Mitochondrial respiration

## Abstract

**Background:**

Diabetic foot ulcer (DFU) is the most devastating complication of diabetes mellitus (DM) and plays a major role in disability and death in DM patients. NADH: ubiquinone oxidoreductase subunit B5 (NDUFB5) plays an important role in maintaining mitochondrial respiration, but whether it is involved in regulating the progression of advanced glycation end products (AGEs)-mediated DFU is still unclear.

**Methods:**

Firstly, the role of AGEs on cell viability, migration, and mitochondrial respiration in human umbilical vein endothelial cells (HUVECs) was explored in vitro. Next, NDUFB5 expression was detected in human samples and AGEs-treated HUVECs, and NDUFB5’s effect on AGEs-induced HUVECs injury and skin wound in diabetic mice was further clarified. In addition, the role of m6A modification mediated by methyltransferase-like 3 (METTL3) in regulating NDUFB5 expression and AGEs-induced HUVECs injury was investigated.

**Results:**

NDUFB5 promoted cell viability, migration, and mitochondrial respiration in AGEs-treated HUVECs, whereas mitochondrial fusion promoter M1 facilitated cell viability, migration, and mitochondrial oxiadative respiration in NDUFB5 knockdown HUVECs. Meanwhile, NDUFB5 promotes skin wound healing in diabetic mice. Besides, METTL3-mediated m6A modification and insulin like growth factor 2 mRNA binding protein 2 (IGF2BP2) enhanced NDUFB5 expression in HUVECs. Furthermore, METTL3 promoted cell viability, migration, and mitochondrial respiration in AGEs-treated HUVECs by increasing NDUFB5.

**Conclusion:**

METTL3-mediated NDUFB5 m6A modification inhibits AGEs-induced cell injury in HUVECs. METTL3 and NDUFB5 might serve as potential targets for DFU therapy in the future.

**Supplementary Information:**

The online version contains supplementary material available at 10.1186/s12967-024-05463-6.

## Background

Diabetes mellitus (DM) is a clinical syndrome mainly characterized by chronic hyperglycaemia caused by absolute or relative insulin insufficiency [1]. Diabetic foot ulcer (DFU) is a general term for foot pain, deep skin ulcers, and gangrene of the extremities caused by a combination of DM, trauma, and infections [2]. DFU is one of the most serious chronic DM complications and causes disability or death in quite a few DM patients [3]. The pathophysiological mechanism of DFU is very complicated, and its pathogenesis mainly involves peripheral vascular disease, neuropathy, infection and skin lesions [4]. In recent years, the relationship between endothelial dysfunction and DFU has received more and more attentions. A large number of studies have shown that diabetic vascular complications caused by endothelial dysfunction play an important role in the pathogenesis of DFU, and are the initial event of the pathophysiological process of DFU [5, 6]. Therefore, a full understanding of the role of endothelial dysfunction in diabetic foot vasculopathy will help to further reveal the pathogenesis of DFU.

Previous studies have revealed that advanced glycation end products (AGEs) played crucial roles in DM occurrence and progression [7]. AGEs promote insulin resistance and contribute to the occurrence and progress of DM-related microvascular and macrovascular complications such as cardiomyopathy [8], nephropathy [9], retinopathy [10], diabetic neuropathy [11] and DFU [12, 13]. Furthermore, endothelial cell morphology and function are impaired due to increased inhibition of proliferation and migration and apoptosis triggered by AGEs [5, 6].

Skin wound healing is a complex mechanism which requires a lot of energy, mainly provided by mitochondrial respiration through the oxidative phosphorylation process [14]. Mitochondrial respiration first involves the oxidation of NADH by the four protein complexes of the respiratory chain to remove electrons it contains. NADH: ubiquinone oxidoreductase subunit B5 (NDUFB5), a key component of Complex I of the electron transport chain, plays an important role in maintaining the mitochondrial respiratory chain [15]. Wu et al. reported that the diabetic skeletal muscle exhibits reduced expression of NDUFB5, which is associated with carbohydrate, energy, and amino acid metabolism [16]. Upregulation of NDUFB5 can accelerate diabetic wound healing [17]. Although NDUFB5 has been implicated in the diabetic wound healing, the mechanisms are not yet clear.

In recent years, studies have revealed that epigenetic modifications induced by environmental stimuli were identified to exert critical roles in the occurrence of DM and serve as therapeutic targets for DM [18]. N6-methyladenosine (m6A) is eukaryotic RNAs’ most commonly-seen chemical modification. m6A modifications are dynamically regulated by methylated transferase (m6A writer) and demethylated transferase (m6A erasers), and their biological effects are determined by the methylated recognition protein (m6A readers) [19, 20]. Methyltransferase-like 3 (METTL3) is the catalytic subunit of the m6A methyltransferase complex and is regarded as the most critical m6A writer [21]. Lin et al. found that high glucose promotes osteoblast ferroptosis through upregulation of METTL3, which in turn promotes the progression of diabetic bone loss [22]. METTL3 expression in pericytes increased after DM, and knockdown of METTL3 inhibited DM-induced pericytes dysfunction and vascular complications [23]. In podocytes, AGEs increased the expression of METTL3, which promoted podocyte death after DM through pro-inflammatory and pro-apoptotic effects, ultimately exacerbating diabetic nephropathy [24]. Meanwhile, METTL3 expression is decreased in DM wounds [25] and METTL3-mediated VEGFC m6A modification enhances VEGFR3-mediated lymphangiogenesis to improve wound healing of DFU [26]. However, the role and underlying mechanisms of METTL3-mediated m6A modification involved in the wound repair of DFUs remain unclear.

In the current study, the effect of AGEs on cell viability, migration, and mitochondrial respiration in human umbilical vein endothelial cells (HUVECs) was explored firstly. Subsequently, NDUFB5’s role in AGEs-induced HUVECs injury and skin wound in diabetic mice was clarified. Besides, we further explored how METTL3-mediated modifications of m6A regulate NDUFB5 expression and HUVECs injury.

## Materials and methods

### Cell culture and transfection

HUVECs were cultured in DMEM (Gibco, Carlsbad, USA) supplementary with 10% FBS (Gibco, Carlsbad, USA). To overexpress NDUFB5 or METTL3, pcDNA3.1(+) plasmid (Addgene, Watertown, USA) was cloned with the coding sequence. To knockdown NDUFB5 or IGF2BP2, siRNAs targeting NDUFB5 or IGF2BP2 was synthesized (Addgene, Watertown, USA). HUVECs were transfected with plasmids or siRNA using Lipofectamine 2000 (Invitrogen, USA) following the manufacture’s protocol. HUVECs transfected with blank pcDNA3.1(+) plasmid or scramble siRNA (siNC) were considered as negative control.

### Experimental grouping

HUVECs were divided into the following five groups: (1) Group 1: Various concentrations of AGEs (100, 200, and 400 µg/mL) were applied to treat HUVECs for 48 h. (2) Group 2: NDUFB5 expression vector was transfected into HUVECs, which were then treated with 200 µg/ml AGEs for 48 h. (3) Group 3: NDUFB5 siRNA was transfected into HUVECs, which were then treated with 10 µM mitochondrial fusion promoter M1 (Sigma-Aldrich, Saint Louis, USA) for 48 h. (4) Group 4: METTL3 expression vector was transfected into HUVECs, which were then treated with 200 µg/ml AGEs for 48 h. (5) Group 5: METTL3 expression vector was used to transfected HUVECs along or not along with NDUFB5 siRNA, then HUVECs were stimulated with 200 µg/ml AGEs for 48 h. (6) Group 5: IGF2BP2 siRNA was used to transfected HUVECs, which were then treated with 200 µg/ml AGEs for 48 h.

### Cell counting kit (CCK)-8 assay

HUVECs (5 × 10^3^ cells/well) were seeded in 96-well plates in DMEM supplementary with 10% FBS and cultured at 37 °C for 18–24 h. After 0, 12, 24, and 48 h of different treatments as described above, HUVECs were incubated with 10 µl/well CCK-8 reagent (Dojindo, Kumamoto, Japan) at 37℃ for 1 h. In the end, the optical density of HUVECs at 450 nm was detected by a microplate reader (Bio-Rad, Hercules, USA).

In vitro**wound healing assay**.

HUVECs (5 × 10^5^ cells/well) were seeded in 6-well culture plates supplemented with DMEM with 10% FBS at 37 °C. When the cells presented a confluence of 100%, the scratches were performed using a 200 µL pipette tip. Afterwards, the cells were washed twice with phosphate buffer saline to remove all detached cells. Next, fresh DMEM medium and different treatments as described above were added to the seeded cells. Wound healing was recorded by photography at 0, 24, and 48 h with an inverted microscope. The distance was measured using ImageJ software version 1.44.

### Extracellular flux analysis

A Seahorse XF-24 Extracellular Flux Analyzer (Seahorse Bioscience, Billerica, USA) was applied to detect oxygen consumption rate (OCR) in HUVECs as previously described [27]. Briefly, HUVECs were cultured in XF-24 culture plates (1 × 10^4^/well, Agilent Technologies, Santa Clara, USA) in an incubator with 5% CO_2_ at 37℃ for 24 h. One hour before assessment, HUVECs were put into an incubator without CO_2_, and XF Base Medium (Agilent Technologies, Santa Clara, USA) was utilized to replace the culture medium. Subsequently, 1 µM oligomycin (ATP synthase inhibitor) was added into “A” well of Seahorse gauging plate, 1.5 µM carbonyl cyanide p-trifluoromethoxy phenylhydrazone (FCCP; uncoupler) was added into “B” well, and then “C” well was instilled with antimycin A (complex III inhibitor; 0.5 µM) and rotenone (complex I inhibitor; 0.5 µM) mixture. The OCR of HUVECs was monitored by the Seahorse XF-24 Extracellular Flux Analyzer.

### Flow cytometry

The reactive oxygen species (ROS) level of the HUVECs was determine by a commercial fluorescent DCFH-DA probe (Beyotime, Shanghai, China). HUVECs were treated with 10 µM DCFH-DA probe at 4 °C for 20 min in the dark, flow cytometry was used to detect the fluorescence of HUVECs at an excitation/emission of 485/530 nm. Moreover, mitochondrial membrane potential (MMP) of HUVECs was detected using a commercial JC-1 assay kit (Beyotime, Shanghai, China) and expressed as JC-1 aggregates (red)/JC-1 monomers (green) fluorescence intensity. Flow cytometry was conducted on a CytoFLEX flow cytometry (BD Biosciences, Franklin Lakes, USA).

### Quantitative real time PCR (qRT-PCR)

TRIzol reagent (Invitrogen, Carlsbad, USA) was applied to extract total RNA. A commercial PrimeScript kit (Takara, Dalian, China) was utilized to reverse transcrive RNA into cDNA. The quantitative RT-PCR was conducted on an ABI 9700 real-time PCR system (Applied Biosystem) using SYBR green (Applied Biosystems, Foster, USA). β-actin was chosen as internal control. The applied primer sequences were as follows: NDUFB5-F: 5ʹ-TCCTGTTCGACACAGTGGAG-3ʹ; NDUFB5-R: 5ʹ-AGGACGGCCATTGTTCTTTCA-3ʹ; METTL3-F: 5ʹ-CCCTATGGGACCCTGACAGA-3ʹ; METTL3-R: 5ʹ-CTGGTTGAAGCCTTGGGGAT-3ʹ; insulin like growth factor 2 mRNA binding protein 2 (IGF2BP2)-F: 5ʹ-GACAGGTCCTGCTGAAGTCC-3ʹ; IGF2BP2-R: 5ʹ-TGTTGACTTGTTCCACATTCTCC-3ʹ; β-actin-F: 5ʹ-AGGATTCCTATGTGGGCGAC-3ʹ; and β-actin-R: 5ʹ-ATAGCACAGCCTGGATAGCAA-3ʹ. The fold-changes of mRNA were calculated by the 2^−ΔΔCT^ method.

### Western blot (WB)

Total protein was extracted by Radio Immunoprecipitation Assay (RIPA) lysis buffer (JRDUN Biotechnogy, Shanghai, China). Proteins were separated by SDS-PAGE and then transferred onto PVDF membranes. Skim milk (5%) was used to block the blots, which were then incubated with primary antibodies against NDUFB5 (ab230215, Abcam, Cambridge, USA), METTL3 (ab195352, Abcam, Cambridge, USA), IGF2BP2 (ab129071, Abcam, Cambridge, USA), and β-actin (66009-1-Ig, Proteintech, Rosemont, USA), followed by incubating with horse radish peroxidase-conjugated secondary antibodies (ZSGB-BIO, Beijing, China). The protein content was assessed by enhanced chemiluminescence system (Bio-Rad, Hercules, USA).

### RNA immunoprecipitation (RIP) assay

RIP was determined by a commercial RIP kit (Millipore, Billerica, USA) following the manufacturer’s instruction. Anti-m6A (#ab208577, Abcam, Cambridge, USA), anti-IGF2BP2 (ab128175, Abcam, Cambridge, USA), or anti-IgG antibody (ab172730, Abcam, Cambridge, USA) were incubated with RNA-protein complexes at 4°C for 1 h. Then, the mixture was added with agarose beads and 50 µL of protein A/G and incubated at 4˚C for 60 min. Subsequently, RIP-wash buffer and RIP-lysis buffer was used to wash the precipitated beads for 10 min and 5 min at 4°C, respectively. Proteinase K was used to treat the immunoprecipitated complex for RNA release. The enrichment of m6A and IGF2BP2 in NDUFB5 3’ untranslated region (UTR) was determined by qRT-PCR.

### mRNA stability detection

Actinomycin D (0.2 mM, GlpBio, Montclair, USA) was utilized to stimulate HUVECs for 0, 2, 4, and 6 h. Total RNA of HUVECs was extracted as described above. The quantitated *NDUFB5* mRNA level was assessed by qRT-PCR.

### Luciferase reporter assay

The 3’UTR of NDUFB5 was cloned in the pGL3-basic vector (Addgene, Watertown, USA). Then, HUVECs were co-transfected with pGL3-NDUFB5-3’ UTR, pRL-TK renilla vectors, METTL3 expression vector, and treated with 200 µg/ml AGEs. After 48 h, the dual-luciferase activities were determined by the Dual-Luciferase Assay System (Promega, Madison, USA).

### Animal models

Beijing Vital River Laboratory Animal Technology Co., Ltd provide us with the male C57BL/6J mice (6-week-old). Mice were injected intraperitoneally with streptozotocin (STZ) citrate buffer (60 mg/kg) to elicit a diabetes model in mice. Blood was drawn from the tail vein, and the glucose level was determined using a glucometer (Accu-check Performa, Roche, Pleasanton, CA, USA). Blood was drawn from the tail vein, and the glucose level was determined using a glucometer (Accu-check Performa, Roche, Pleasanton, CA, USA). AGEs were determined using an AGE assay kit (ab238539, Abcam, Cambridge, USA) according to the manufacturers’ instructions. Two weeks after STZ injection, the blood glucose, AGEs and body weight were measured. Mice with glucose levels higher than 16.70 mmol/L were considered as diabetic mice. A scissor was used to make skin wounds (1.3 × 1.3 cm) on the mid-back of mice at 14 days after STZ administration (day 0). A total of 200 µL NDUFB5-expressing adenovirus vector (Ad-NDUFB5) or blank adenovirus vector was injected at the sites of skin wound at 16 days after STZ administration (day 2) (*n* = 6 per group). Mice in the control group were injected with blank adenovirus vector at the sites of skin wound at 16 days after citrate buffer administration (*n* = 6 per group). Wounds were tested at day 0, 7, and 14, respectively.

### Hematoxylin and eosin (HE) staining

Wound tissues were fixed in 4% paraformaldehyde and HE staining was performed as conducted described previously [28]. The digital images were captured by an Olympus DP71 camera (Olympus Corporation, Japan). Blinded operators performed the image analyses.

### Assessment of oxidative stress in wound tissues

The malondialdehyde (MDA; A003), glutathione peroxidase (GSH-PX; A005), and superoxide dismutase (SOD; A001, all from Nanjing Jiancheng Bioengineering Institute) were measured in wound tissues. Following the manufacturer’s instructions, all indicators were measured using a microplate reader with the appropriate detection kits.

### Clinical samples of DFU

DFU patients (30 cases) and non-diabetic patients with trauma (15 cases) in Hospital were included and their skin samples were collected. Electronic medical records of all patients were reviewed. All data were collected from hospital information system. The clinical information of the patients with DFU consisted of age, sex, and body mass index (BMI). The severity of the foot ulcers was classified on the basis of diabetic ulcer severity score (DUSS). Fifteen patients are diagnosed with DUSS 1–2 and 15 patients are with DUSS 3–4. Baseline laboratory data including fasting blood glucose (FBG), glycosylated hemoglobin (HbA1c), Hb, platelet (PLT), white blood cell (WBC) count, serum albumin, C-reactive protein (CRP) and AGEs were collected and shown in Table 1. Informed consent was gained from all participants. This study was authorized by the Ethics Committee of Qingpu Branch of Zhongshan Hospital, affiliated to Fudan University (No.Qingyi2021-20).

**Table 1:** Baseline demographic and laboratory data between the DFU patients and non-diabetic patients with trauma as control

	Control (*n* = 15)	DFU (*n* = 30)	*P* value
**Age**	62.3 ± 8.37	64.7 ± 7.71	0.2953
**Gender (male %)**	8 (53.3%)	17 (56.7%)	0.8320
**BMI**,** kg/m**^**2**^	20.9 ± 1.36	21.4 ± 1.50	0.2334
**FBG**,** mmol/L**	4.87 ± 0.50	9.61 ± 2.37	< 0.001
**HbA1c**,** %**	4.58 ± 0.75	9.60 ± 0.72	< 0.001
**Hb**,** g/L**	142.2 ± 9.37	114.1 ± 12.9	< 0.001
**PLT**,** ×10**^**9**^**/L**	192.8 ± 59.6	167.6 ± 44.2	0.1895
**Albumin**,** g/L**	43.8 ± 4.65	36.2 ± 3.02	< 0.001
**WBC count**,** ×10**^**9**^**/L**	6.97 ± 1.47	7.84 ± 1.74	0.1224
**CRP**,** mg/L**	2.31 ± 1.25	22.3 ± 11.2	< 0.001
**AGEs**,** µg/mL**	22.2 ± 4.54	36.2 ± 8.9	< 0.001

BMI, body mass index. FBG, fasting blood glucose. HbA1c, glycosylated hemoglobin. Hb, hemoglobin. PLT, platelet. WBC, white blood cell. CRP, C-reactive protein. Differences between two groups were assessed using the Mann–Whitney U test for continuous variables and chi-square test for categorial variables

### Statistical analysis

All experiments were conducted at least three times independently. All statistical analyses were performed by GraphPad Prism 8.4.2. All data were subjected to normal distribution and homogeneous variance tests, and data conforming to normal distribution were expressed in the form of mean ± standard deviation. Independent sample t-test was used for comparison between two groups, and data between multiple groups were compared by one-way ANOVA, followed by Tukey post hoc test. *P* < 0.05 was considered statistical significance.

## Results

### AGEs inhibit cell viability, migration, and mitochondrial respiration of HUVECs

**Fig. 1:**
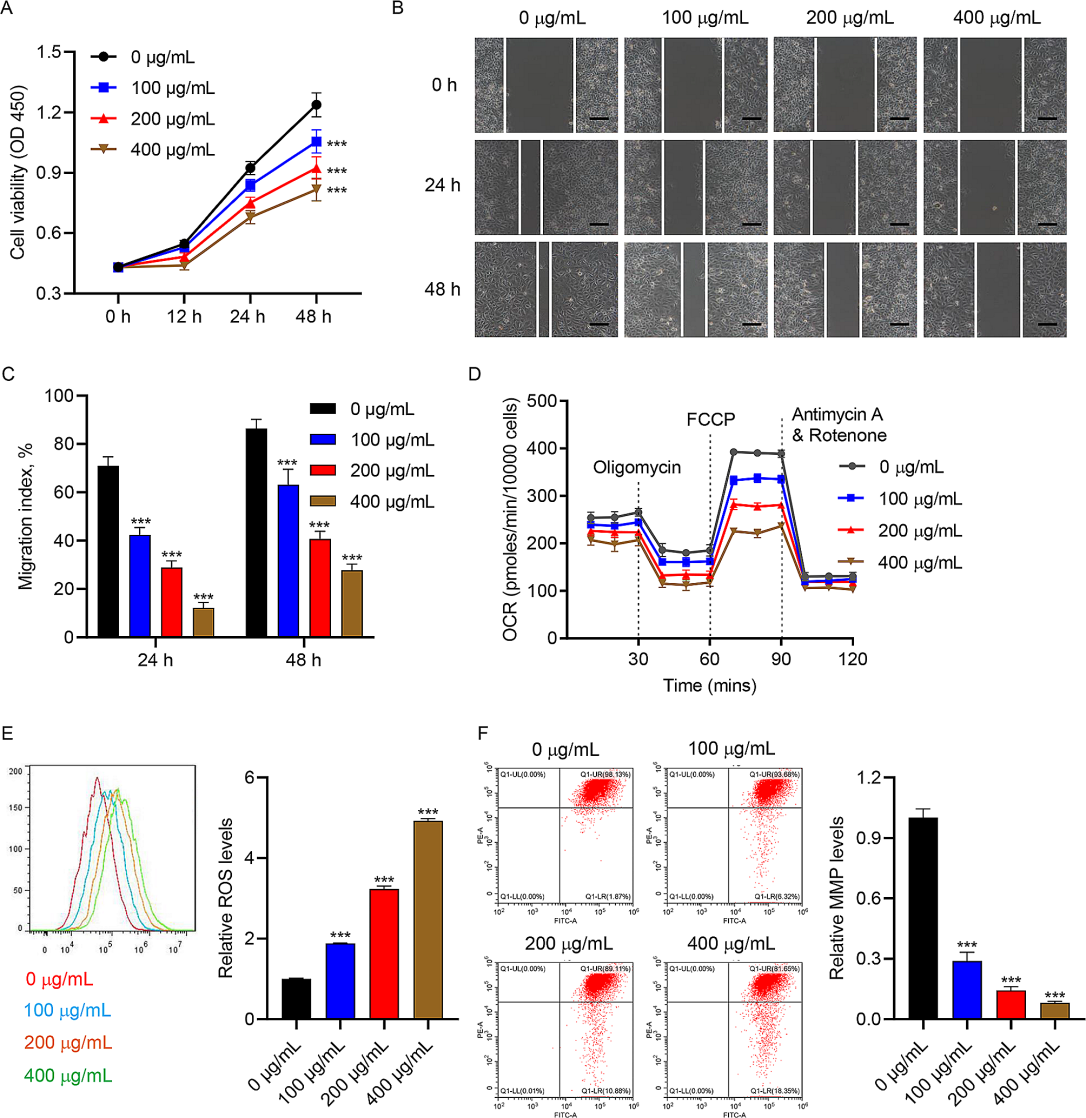
AGEs inhibit cell viability, migration, and mitochondrial respiration in HUVECs. Different concentrations of AGEs were used to treat HUVECs for 48 h. (**A**) Cell viability of HUVECs was detected by CCK-8 assay. (**B, C**) In vitro wound healing assay of HUVECs (scale bar, 100 μm). (**D**) The OCR of HUVECs was assessed by Seahorse XF-24 Extracellular Flux Analyzer. Flow cytometry analysis of (**E**) ROS and (**F**) MMP levels in HUVECs. ^***^P < 0.001 vs. 0 µg/mL group

First, we investigated the roles of AGEs in cell viability, migration and mitochondrial respiration of HUVECs. CCK-8 assay showed that cell viability of HUVECs was gradually decreased with the increase of AGEs concentration (Fig. 1A). AGEs inhibited HUVECs migration in a concentration-dependent way (Fig. 1B-C). Besides, AGEs inhibited cellular OCR (Fig. 1D), increased ROS generation (Fig. 1E) and down-regulated MMP levels (Fig. 1F) in HUVECs. These results suggested that AGEs inhibited cell viability, migration and mitochondrial respiration in HUVECs.

### NDUFB5 promotes cell viability, migration, and mitochondrial respiration in AGEs-treated HUVECs

**Fig. 2:**
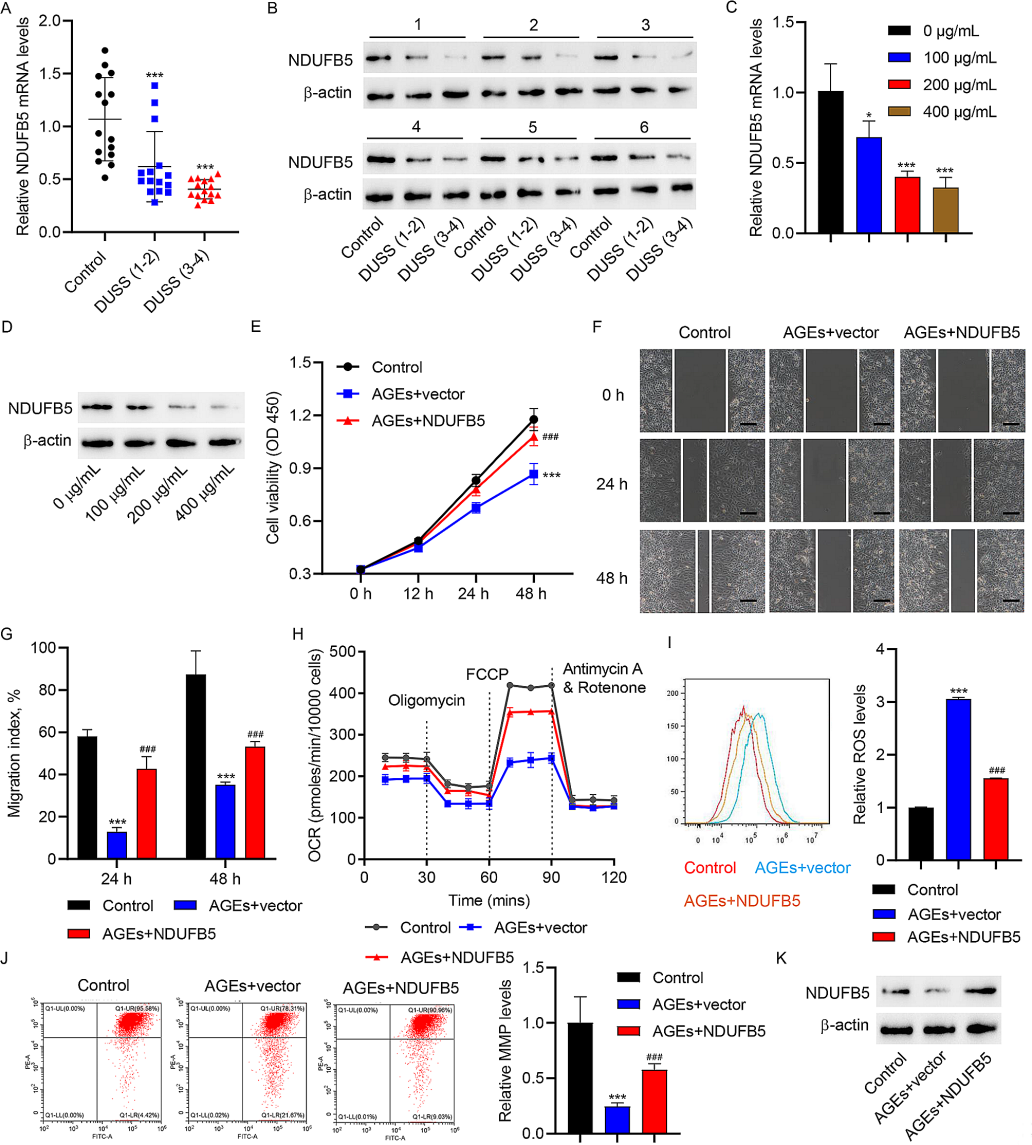
NDUFB5 promotes cell viability, migration, and mitochondrial respiration in AGEs-treated HUVECs. Ulcer tissue samples from DFU patients of DUSS grade 1–2 or 3–4 and skin tissues from normal trauma patients were collected. (**A**) qRT-PCR analysis and (**B**) WB analysis of NDUFB5 expression in tissues. Different concentrations of AGEs were applied to stimulate HUVECs for 48 h. (**C**) qRT-PCR analysis and (**D**) WB analysis of NDUFB5 expression in HUVECs. HUVECs were transfected with NDUFB5 expression vector or blank vector and stimulated with 200 µg/mL AGEs or vehicle for 48 h, and HUVECs in control group were transfected with blank vector and stimulated with vehicle. (**E**) Cell viability of HUVECs was detected by CCK-8 assay. (**F, G**) In vitro wound healing assay of HUVECs (scale bar, 100 μm). (**H**) The OCR of HUVECs was assessed by Seahorse XF-24 Extracellular Flux Analyzer. Flow cytometry analysis of (**I**) ROS and (**J**) MMP levels in HUVECs. (**K**) WB analysis of NDUFB5 expression in HUVECs. ^*^P < 0.05, ^***^P < 0.001 vs. 0 µg/mL or control group. ^###^*P* < 0.001 vs. AGEs + vector group

We next clarified NDUFB5’s effects on cell viability, migration, and mitochondrial respiration of HUVECs. First, we collected skin samples from normal subjects and DFU patients, as confirmed by qRT-PCR and WB, NDUFB5 were significantly decreased in the skin lesion tissues of DFU patients as compared to normal subjects (Fig. 2A-B). As DFU progressed, NDUFB5 expression was further reduced in skin lesions of DFU patients (Fig. 2A-B). In vitro, AGEs inhibited NDUFB5 expression in HUVECs in a concentration-dependent manner (Fig. 2C-D). After overexpression of NDUFB5 in HUVECs (Figure S1A), AGEs’ inhibitory effects on cell viability (Fig. 2E), migration (Fig. 2F-G), OCR (Fig. 2H), MMP levels (Fig. 2J) and NDUFB5 expression (Fig. 2K) were abolished, whereas AGEs-induced ROS generation in HUVECs was suppressed (Fig. 2I). Data above implied that NDUFB5 boosted cell viability, migration, and mitochondrial respiration in AGEs-treated HUVECs.

### **Mitochondrial fusion promotes cell viability**,** migration**,** and mitochondrial respiration in HUVECs with NDUFB5 knockdown**

**Fig. 3:**
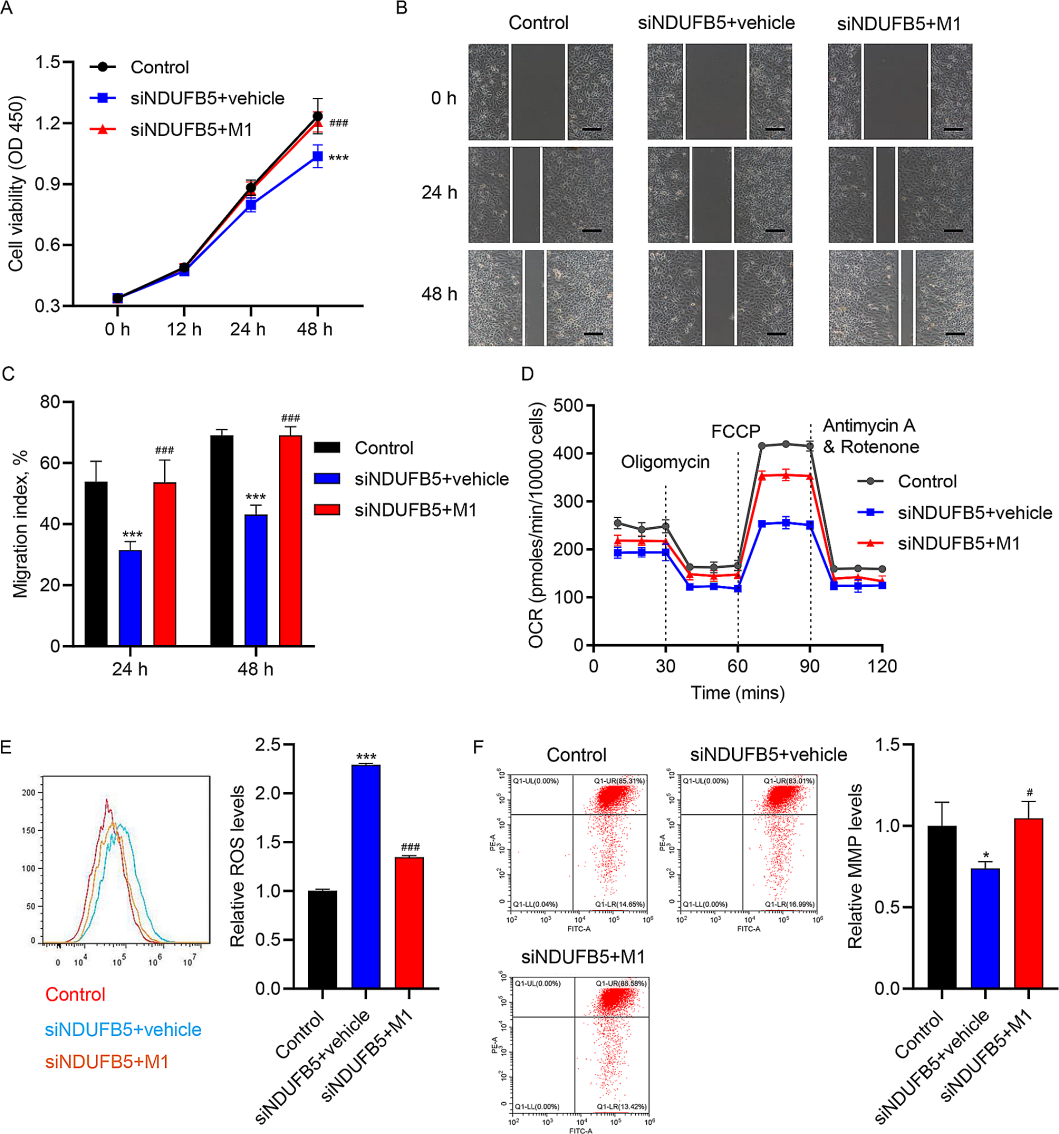
Mitochondrial fusion promotes cell viability, migration, and mitochondrial respiration in NDUFB5 siRNA transfected HUVECs. HUVECs were transfected with siNDUFB5 or siNC and treated with 10 µM mitochondrial fusion promoter M1 or vehicle for 48 h, and HUVECs in control group were transfected with siNC and treated with vehicle. (**A**) Cell viability of HUVECs was detected by CCK-8 assay. (**B, C**) In vitro wound healing assay of HUVECs (scale bar, 100 μm). (**D**) The OCR of HUVECs was assessed by Seahorse XF-24 Extracellular Flux Analyzer. Flow cytometry analysis of (**E**) ROS and (**F**) MMP levels in HUVECs. ^*^P < 0.05, ^***^P < 0.001 vs. control group. ^#^*P* < 0.05, ^###^*P* < 0.001 vs. siNDUFB5 + vehicle group

Considering that NDUFB5 acts as a crucial subunit for maintaining NADH dehydrogenase assembly and function [29], NDUFB5 may affect the function of HUVECs by targeting mitochondria. Upon NDUFB5 knockdown in HUVECs (Figure S1B), the cell viability of HUVECs was significantly reduced (Fig. 3A), accompanied by down-regulation of migration (Fig. 3B-C), OCR (Fig. 3D) and MMP levels (Fig. 3F) as well as up-regulation of ROS generation (Fig. 3E). Mitochondrial fusion is an important mechanism to maintain mitochondrial homeostasis [30]. Mitochondrial fusion promoter M1 ameliorated siNDUFB5-induced decreases in cell viability (Fig. 3A), migration (Fig. 3B-C), OCR (Fig. 3D) and MMP levels (Fig. 3F), while inhibiting siNDUFB5-induced ROS production (Fig. 3E) in HUVECs. These data demonstrated that NDUFB5 might bolster cell viability, migration, and mitochondrial respiration in HUVECs by inducing mitochondrial fusion.

### METTL3-mediated m6A modification and IGF2BP2 may enhance NDUFB5 expressions in HUVECs

**Fig. 4:**
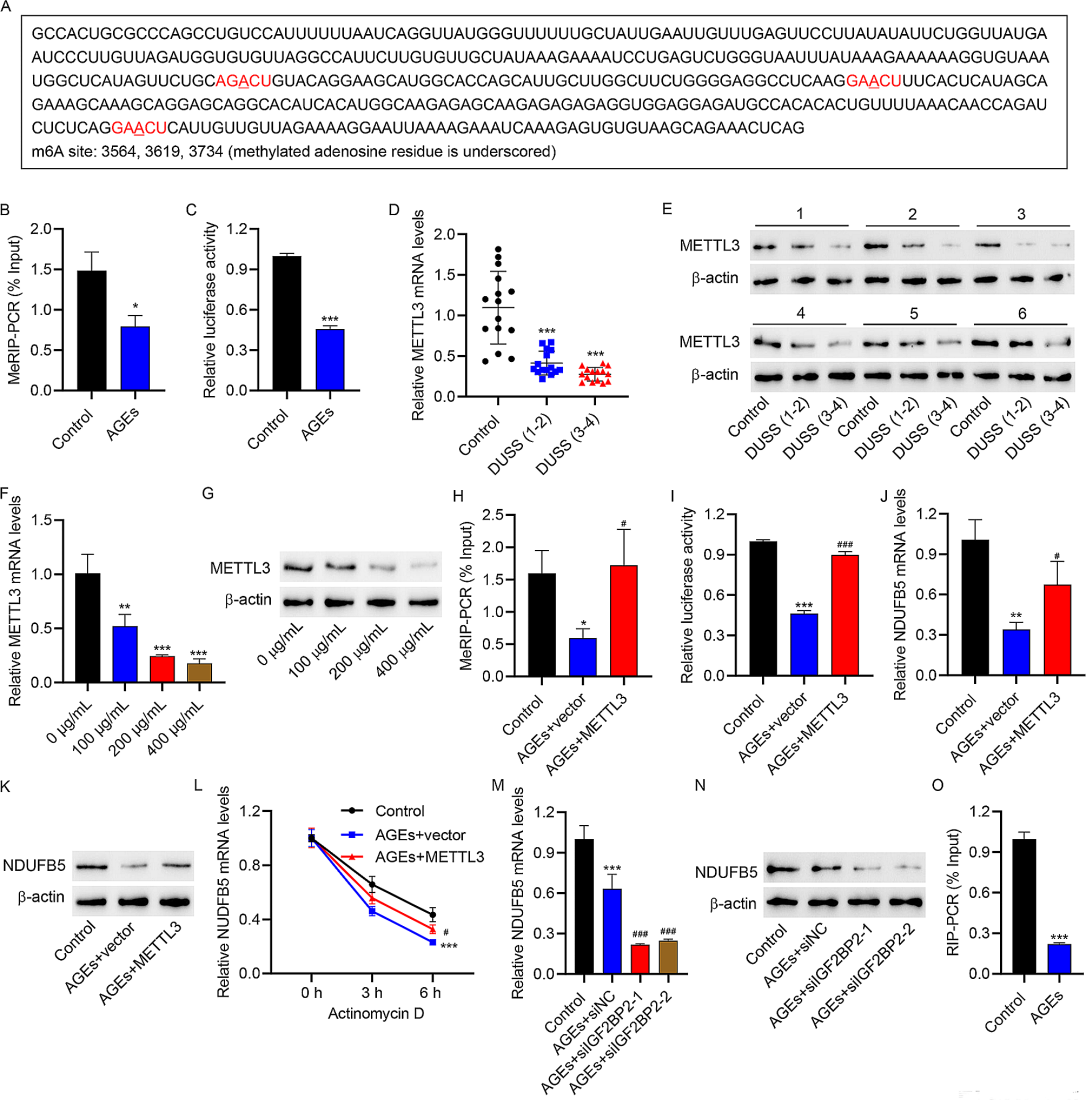
m6A modificated by METTL3 and IGF2BP2 may enhance HUVECs NDUFB5 expression. (**A**) The predicted m6A sites in NDUFB5 with use of SRAMP. AGEs (200 µg/mL) was used to stimulate HUVECs for 48 h. (**B**) MeRIP was used to detect the m6A level of NDUFB5 3’UTR in HUVECs. (**C**) Dual luciferase reporter gene assay of NDUFB5 3’UTR activity in HUVECs. Ulcer tissue samples from DFU patients of DUSS grade 1–2 or 3–4 and skin tissues from normal trauma patients were collected. (**D**) qRT-PCR analysis and (**E**) WB analysis of METTL3 expression in tissues. Different concentrations of AGEs were applied to stimulate HUVECs for 48 h. (**F**) qRT-PCR analysis and (**G**) WB analysis of METTL3 expression in HUVECs. HUVECs were transfected with METTL3 expression vector or blank vector and stimulated with 200 µg/ml AGEs or vehicle for 48 h, and HUVECs in control group were transfected with blank vector and stimulated with vehicle. (**H**) MeRIP was applied to assess the m6A level of NDUFB5 3’UTR in HUVECs. (**I**) Dual luciferase reporter gene assay of NDUFB5 3’UTR activity in HUVECs. (**J**) qRT-PCR analysis and (**K**) WB analysis of NDUFB5 expression in HUVECs. (**L**) NDUFB5 mRNA stability analysis in actinomycin D-treated HUVECs. HUVECs were transfected with siIGF2BP2 or siNC and stimulated with 200 µg/ml AGEs or vehicle for 48 h, and HUVECs in control group were transfected with siNC and stimulated with vehicle. (**M**) qRT-PCR analysis and (**N**) WB analysis of NDUFB5 expression in HUVECs. (**O**) AGEs (200 µg/mL) was used to stimulate HUVECs for 48 h. RIP assay of enrichment of NDUFB5 3’UTR by anti-IGF2BP2. ^*^P < 0.05, ^**^P < 0.01, ^***^P < 0.001 vs. control or 0 µg/mL group. ^#^*P* < 0.05, ^###^*P* < 0.001 vs. AGEs + vector or AGEs + siNC group

SRAMP analysis revealed abundant m6A modification sites in NDUFB5 3’UTR (Fig. 4A). To investigate whether m6A modification regulate NDUFB5 expression in HUVECs, we examined the m6A level and activity of NDUFB5 3’UTR in HUVECs. The m6A level of NDUFB5 3’UTR was significantly reduced in AGEs-treated HUVECs (Fig. 4B), accompanied by a decrease in NDUFB5 3’UTR activity (Fig. 4C). Previous studies have shown that METTL3 expression is decreased in DM wounds [25] and METTL3-mediated VEGFC m6A modification enhances VEGFR3-mediated lymphangiogenesis to improve wound healing of DFU [26]. Thus, we speculate that METTL3 may mediate NDUFB5 m6A modification to improve wound healing of DFU. Compared with normal subjects, METTL3 expression in skin lesion tissues of DFU patients was gradually decreased with the progression of DFU (Fig. 4D-E). AGEs also inhibited METTL3 expression of HUVECs in a concentration-dependent manner (Fig. 4F-G). After METTL3 overexpression (Figure S1C), the m6A level of NDUFB5 3’UTR (Fig. 4H), NDUFB5 3’UTR activity (Fig. 4I) and NDUFB5 expression (Fig. 4J-K) were significantly increased in AGEs-treated HUVECs. Meanwhile, METTL3 overexpression promoted the stability of NDUFB5 mRNA in AGEs-treated HUVECs (Fig. 4L). IGF2BP2 is an important m6A reader that promotes the stability of target RNAs by recognizing m6A modifications [31]. Studies have confirmed that IGF2BP2 was an essential modulator in metabolism and the occurrence and development of DM [32, 33]. After IGF2BP2 knockdown (Figure S1D), NDUFB5 level was further reduced in AGEs-treated HUVECs cells (Fig. 4M-N). RIP confirmed that the binding between IGF2BP2 and the NDUFB5 3’UTR was decreased in AGEs-treated HUVECs cells (Fig. 4O). The above data indicated that both METTL3-mediated m6A modification as well as IGF2BP2 played important roles in upregulating NDUFB5 in AGEs-treated HUVECs.

#### METTL3 promotes cell viability, migration, and mitochondrial respiration in AGEs-treated HUVECs by increasing NDUFB5

**Fig. 5:**
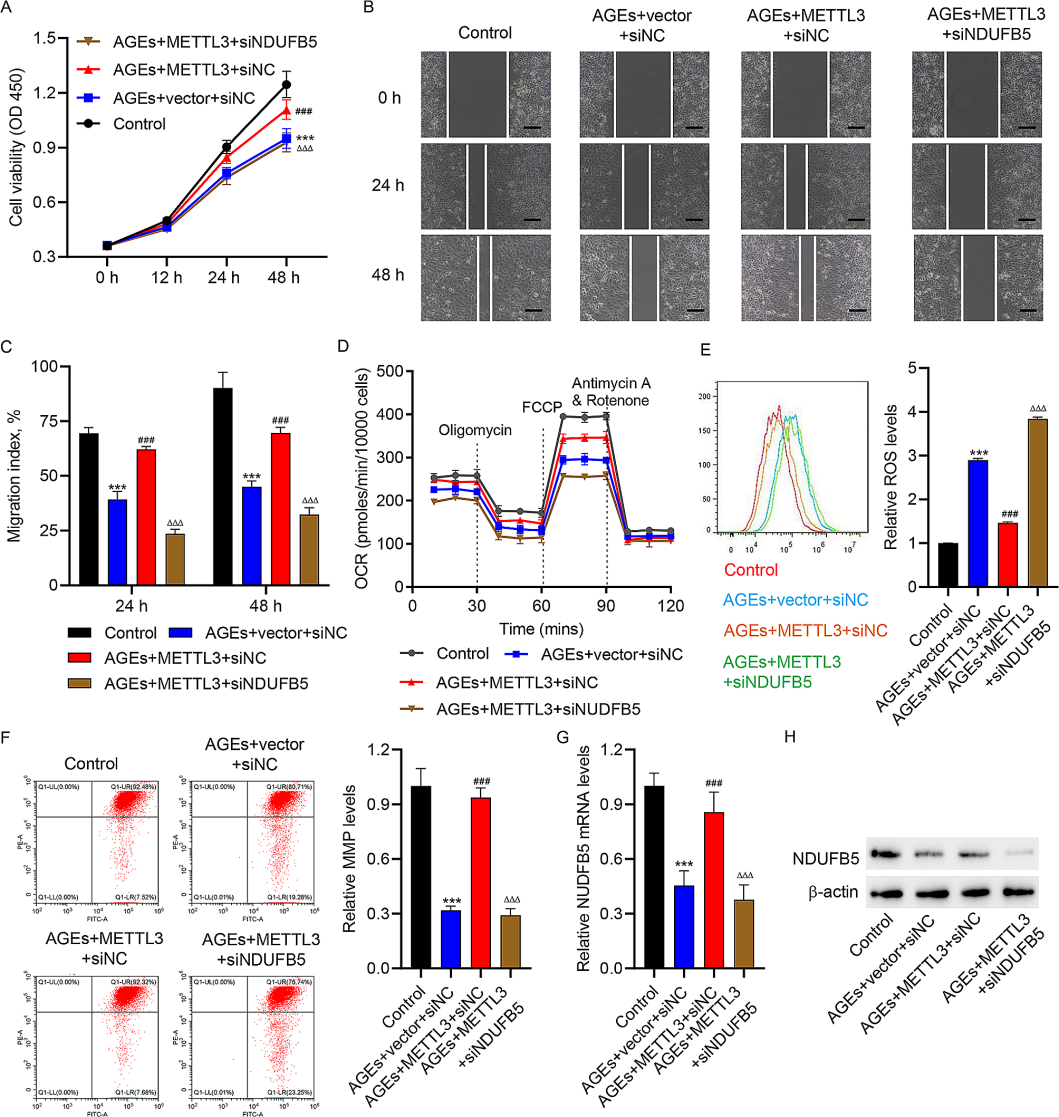
NDUFB5 knockdown inhibits METTL3’s effect on the cell viability,** migration**,** and mitochondrial respiration in AGEs-treated HUVECs.** METTL3 expression vector or blank vector was used to transfected HUVECs along or not along with NDUFB5 siRNA or siNC, then HUVECs were stimulated with 200 µg/ml AGEs or vehicle for 48 h, and HUVECs in control group were transfected with blank vector, siNC and stimulated with vehicle. (**A**) Cell viability of HUVECs was detected by CCK-8 assay. (**B, C**) In vitro wound healing assay of HUVECs (scale bar, 100 μm). (**D**) The OCR of HUVECs was assessed by Seahorse XF-24 Extracellular Flux Analyzer. Flow cytometry analysis of (**E**) ROS and (**F**) MMP levels in HUVECs. (**G**) qRT-PCR analysis and (**H**) WB analysis of NDUFB5 expression in HUVECs. ^***^P < 0.001 vs. control group. ^###^*P* < 0.001 vs. AGEs + vector + siNC group. ^ΔΔΔ^*P* < 0.001 vs. AGEs + METTL3 + siNC group

Since METTL3 upregulates NDUFB5 in HUVECs, we wondered whether the function of HUVECs was modulated by METTL3/NDUFB5 axis or not. Upon METTL3 overexpression, AGEs-induced reduction in cell viability (Fig. 5A), impaired migration (Fig. 5B-C), decrease of OCR (Fig. 5D), MMP reduction (Fig. 5F), increase in ROS generation (Fig. 5E), and NDUFB5 down-regulation (Fig. 5G-H) were abolished in HUVECs. Nevertheless, NDUFB5 knockdown markedly weakened the protective effects of METTL3 on cell viability, migration, and mitochondrial respiration in AGEs-treated HUVECs (Fig. 5A-H). These results implied that METTL3 ameliorated the impairment of cell viability, migration, and mitochondrial respiration in HUVECs caused by AGEs via increasing NDUFB5.

### NDUFB5 promotes skin wound healing in diabetic mice

**Fig. 6:**
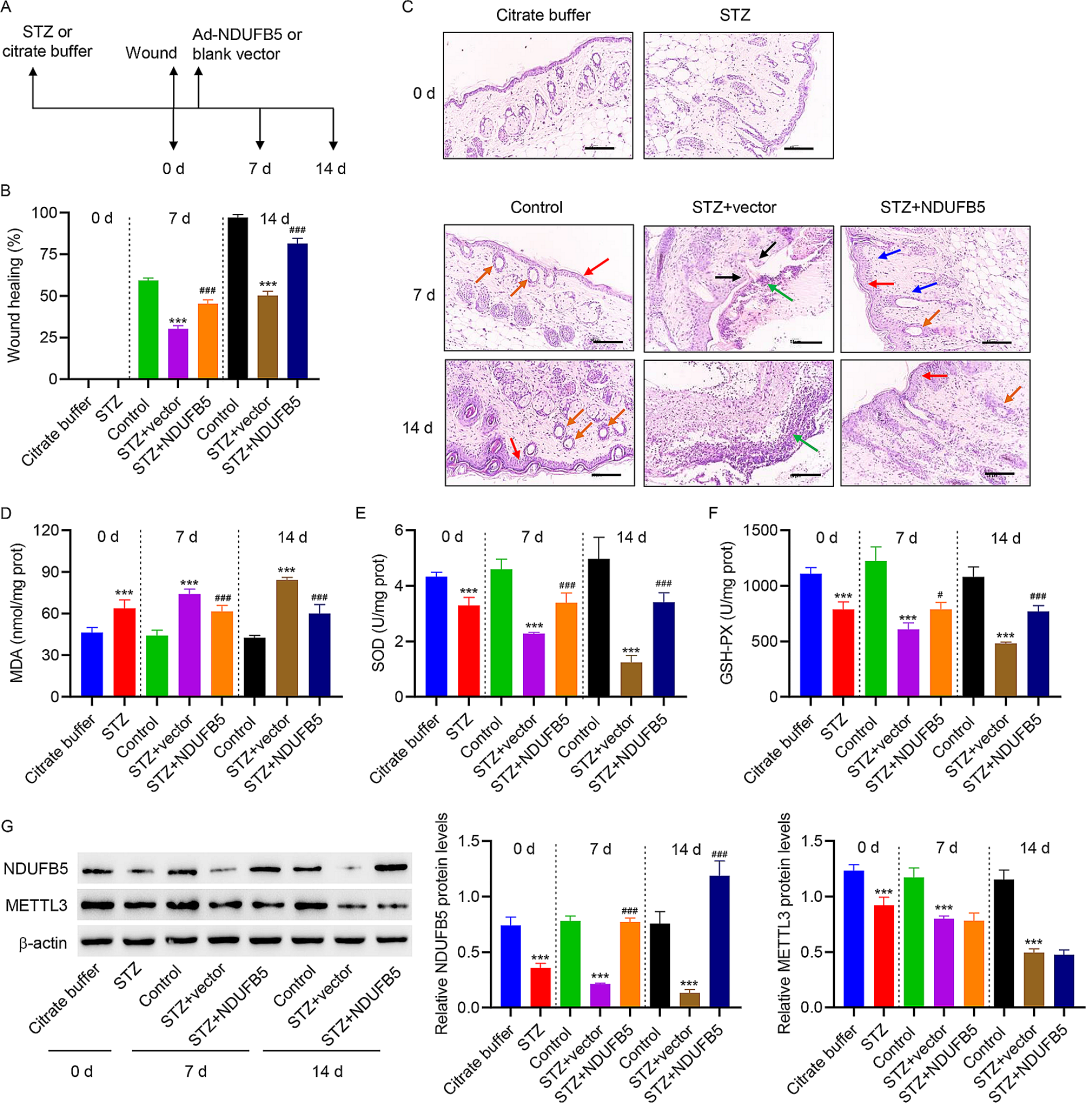
NDUFB5 promotes skin wound healing in diabetic mice. (**A**) STZ (60 mg/kg) was applied to intraperitoneally inject mice to construct DM model. Skin wounds was produced by a biopsy perforator 14 days after STZ injection (marked as day 0). After 16 days of STZ administration (marked as day 2), mice were injected with Ad-NDUFB5 or blank adenovirus vector in the wounds to overexpress NDUFB5. Mice in the control group were injected with blank adenovirus vector at the sites of skin wound at 16 days after citrate buffer administration. (**B**) Wound healing rate was calculated. (**C**) HE staining of the skin tissues from different groups (scale bar, 100 μm). Control mice show surface epidermis (red arrow) and the underlying dermis with hair follicles (orange arrow). In STZ-induced diabetic mice, epidermal tongue (black arrow) is seen at the proliferating creepy epidermis, and some wounds show an abnormal irregular scab (green arrow) covering the wound area. Ad-NDUFB5-injected diabetic mice show intact epidermis (red arrow) covering the wound area, and the dermis appears normal with frequent hair follicles (orange arrow) and blood vessel (blue arrow). The levels of (**D**) MDA, (**E**) SOD, and (**F**) GSH-PX in skin tissues. (**G**) WB analysis of NDUFB5 and METTL3 level in skin tissues. ^***^P < 0.001 vs. citrate buffer or control group. ^#^*P* < 0.05, ^###^*P* < 0.001 vs. STZ + vector group

To elucidate NDUFB5’s effect on wound healing in diabetic mice, we constructed an STZ-induced DM model and produced skin wounds 14 days after STZ injection. The blood glucose levels in citrate buffer and STZ-induced diabetic mice were 7.72 ± 0.60 mmol/L and 26.17 ± 1.42 mmol/L, respectively. The serum AGEs levels in citrate buffer and STZ-induced diabetic mice were 20.13 ± 2.53 µg/mL and 42.37 ± 5.05 µg/mL, respectively. The body weight in citrate buffer and STZ-induced diabetic mice were 22.97 ± 0.53 g and 19.43 ± 1.02 g, respectively. These data indicated that the diabetic mice model was successfully constructed. After 16 days of STZ administration, mice were injected with Ad-NDUFB5 or blank adenovirus vector in the wounds to overexpress NDUFB5 (Fig. 6A). As shown in Fig. 6B, wound healing in STZ-induced diabetic mice was slower compared to control. However, STZ-induced diabetic mice injected with Ad-NDUFB5 accelerated wound healing (Fig. 6B), suggesting that NDUFB5 overexpression promotes wound healing. HE-stained skin slices from the control group showed normal covering epidermis and underlying dermis with notable hair follicles (Fig. 6C). In STZ-induced diabetic mice, epidermal tongue was seen at the proliferating creepy epidermal edges, and abnormal irregular scab covered the wound area. At the wound area, dermis was devoid of hair follicles (Fig. 6C). The examined skin sections of the Ad-NDUFB5 treated group showed intact epidermis that completely covered the wound area, and the dermis was normal with frequent hair follicles and blood vessel (Fig. 6C). These results suggested that Ad-NDUFB5 injection in STZ-induced diabetic mice results in accelerated wound repair. Furthermore, the levels of MDA, GSH-PX, and SOD were determined to evaluate oxidative stress. As shown in Fig. 6D, MDA levels in STZ-induced diabetic mice was higher compared to control. However, STZ-induced diabetic mice injected with Ad-NDUFB5 decreased MDA levels. Moreover, SOD and GSH-PX levels in STZ-induced diabetic mice was lower compared to control (Fig. 6E-F). However, STZ-induced diabetic mice injected with Ad-NDUFB5 increased SOD and GSH-PX levels. After STZ treatment, NDUFB5 and METTL3 expressions in wound tissues were significantly reduced (Fig. 6G). These results confirmed that NDUFB5 promoted skin wound healing in diabetic mice.

## Discussion

**Fig. 7:**
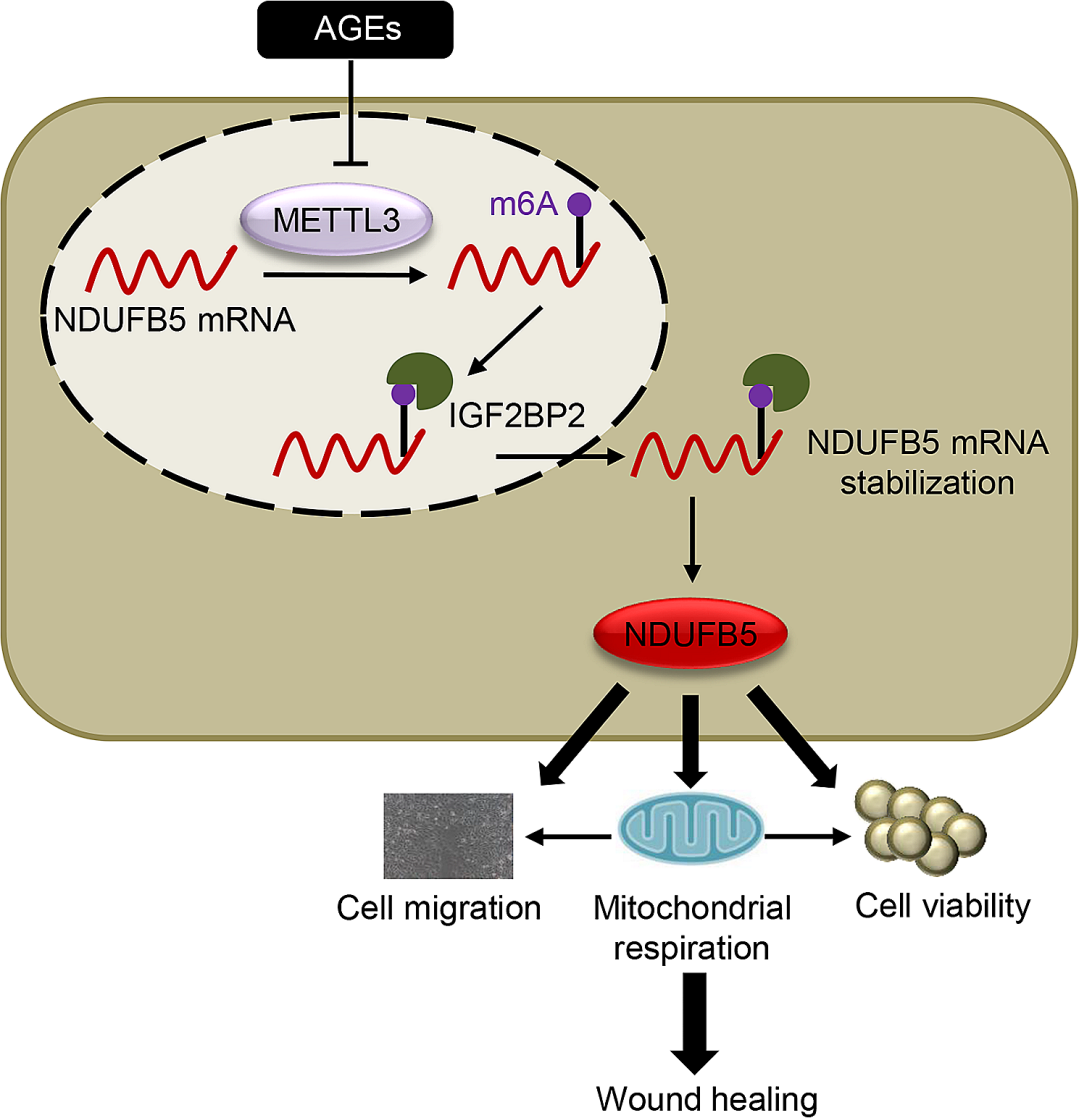
Diagram of the mechanisms underlying METTL3-mediated NDUFB5 m6A modification regulation of cell migration and mitochondrial respiration in diabetic foot ulcer wound healing

DM is a chronic disease severely jeopardizing human health. DFU is a common and most serious complication of DM, which lacks effective treatment. Therefore, shedding light on the pathogenesis of DFU and searching for effective therapeutic targets is of great significance. In the present study, NDUFB5 was found to promote cell viability, migration, and mitochondrial respiration in AGEs-treated HUVECs, which may be associated with mitochondrial fusion. Besides, NDUFB5 promoted skin wound healing in diabetic mice, and METTL3-mediated m6A modification as well as IGF2BP2 played important roles in upregulating NDUFB5 in HUVECs. Furthermore, METTL3 ameliorated the injury of cell viability, migration, and mitochondrial respiration in HUVECs caused by AGEs via upregulating NDUFB5 (Fig. 7). Therefore, METTL3 and NDUFB5 may be potential targets for future DFU therapy.

Previous studies have found that AGEs induced the development of DM through two mechanisms: direct trapping/cross-linking proteins, and interacting with cell surface receptors. AGEs can modulate the function of target cells by binding to RAGE, G-protein-coupled receptors, Toll-like receptors, scavenger receptors, etc., among which the most important is RAGE [34, 35]. Upon binding of AGEs to RAGE, downstream NF-κB and MAPK signaling pathways activation promotes cellular synthesis of ROS, as well as pro-inflammatory and pro-fibrotic factors, such as VCAM-1, PAI-1, MCP-1, MMP-2, etc. [36, 37]. These factors contribute to thrombosis, atherosclerosis, vascular calcification, and vascular plaque accumulation [38, 39]. In addition, accumulation and cross-linking of AGEs on long-lived matrix proteins such as collagen and elastin also lead to atherosclerosis and endothelial dysfunction, disrupting extracellular matrix-cell interactions [40–42]. It has been found that AGEs-induced protein modifications are not only associated with cardiovascular disease, but also closely associated with DM-induced nephropathy, neuropathy, and retinopathy [43]. We found that AGEs inhibited cell viability, migration, mitochondrial respiration, and MMP levels, while promoting ROS synthesis in vascular endothelial cells, suggesting that AGEs also inhibit the normal function of vascular endothelial cells. Similarly with our results, Wu et al. found that AGEs promoted apoptosis in vascular endothelial cells, along with proinflammatory factors and oxidative stress levels [44]. In addition, AGEs have been found to increase vascular endothelial cell permeability [4] and senescence [45]. Thus, AGEs exert vital functions in promoting vascular endothelial injury and thus DFU after DM.

NDUFB5 is one of the 45 subunits of NADH dehydrogenase. NADH dehydrogenase, also known as complex I, is the main entry point for electron transfer in the mitochondrial respiratory chain and the largest of the four complexes of the respiratory chain [46]. NDUFB5, although not directly involved in the catalytic action of NADH dehydrogenase, is a subunit essential for the assembly and normal function of NADH dehydrogenase [47]. Little is known about the function of NDUFB5, and its effects on the vascular endothelium as well as DM are even less understood. Given the critical role of NDUFB5 in the mitochondrial respiratory chain, we hypothesized that NDUFB5 is likely to ameliorate AGEs-induced endothelial cell damage by improving cellular energy supply. In addition, we found that mitochondrial fusion promoter M1 successfully reversed NDUFB5 knockdown-induced endothelial cell injury. An upregulation of mitochondrial fusion increases mitochondrial oxidative capacity and is pivotal in the management of cellular stress through cross-complementation [48]. Mitochondrial fusion is also an important step in mitochondrial biogenesis and an important mechanism for maintaining mitochondrial energy supply and resisting external energy stress [49] and this fusion process helps give the cristae its curvature, enhancing oxidative phosphorylation by allowing for a greater distribution of oxidative phosphorylation complexes [50], decreasing the diffusion of substrates and electron transfer and further improving mitochondria-to-mitochondria communication [51]. Our results suggested that mitochondrial fusion was also a potential mechanism by which NDUFB5 exerted endothelial protection.

m6A is eukaryotic RNAs’ most frequent chemical modification. m6A modifications tend to occur in RRACH (R: purine, H: non-guanine base) sequences, which often located near the stop codons of mRNAs and enriched in the 3’UTR and long internal exons [52, 53]. Our data revealed that the m6A modification of the NDUFB5 3’UTR was significantly reduced in HUVECs treated with AGEs, which drove us to further explore the methyltransferase that mediates NDUFB5 m6A modification. METTL3 is the first m6A methyltransferase to be discovered and is the catalytic core component of the m6A methyltransferase complex [54]. Previous studies have reported that high glucose and AGEs induce METTL3 expression to promotes the progression of diabetic bone loss [22], diabetic retinopathy [23], and diabetic nephropathy [24]. Contrary to these studies, our data exhibted a gradual decrease in METTL3 level in skin lesion tissues after DFU, and AGEs decreased METTL3 expression in HUVECs. METTL3 overexpression ameliorated the damage of AGEs on HUVECs. We speculated that this might be related to the fact that METTL3 plays different roles in different cells and different complications after DM. Therefore, targeting METTL3 for DFU therapy in the future needs to consider its conflicting roles in different tissues and organs. Local METTL3 overexpression in skin tissues, rather than systemic overexpression of METTL3, would be an optimal choice.

The biological effects of m6A modifications are determined by m6A readers, which affect almost all metabolic processes such as alternative splicing, nucleoplasm translocation, stabilization, degradation, and translation of mRNA [55]. Our results showed that overexpression of METTL3 enhanced m6A modification of the NDUFB5 3’UTR in HUVECs, accompanied by an increase in NDUFB5 expression and NDUFB5 mRNA stability. Thus, m6A readers promote the stability of NDUFB5 mRNA by recognizing its m6A modification. IGF2BP2 is a classical m6A reader that promotes the stability of target mRNAs [32]. Several studies have confirmed that the expression level and single nucleotide polymorphisms of IGF2BP2 are directly associated with the occurrence and progression of DM [33]. Our results showed that there was binding between IGF2BP2 and NDUFB5 mRNA in HUVECs, and NDUFB5 expression was reduced after knockdown of IGF2BP2. Thus, following METTL3-mediated m6A modification NDUFB5, IGF2BP2 likely promoted NDUFB5 mRNA stability by recognizing its m6A modification, which in turn promoted vascular endothelial NDUFB5 expression.

## Conclusions

In conclusion, our study confirmed the inhibitory effect of NDUFB5 on AGEs-induced vascular endothelial injury, as well as the critical role of METTL3-mediated m6A modification in upregulating NDUFB5 and ameliorating AGEs-associated vascular endothelial dysfunction. In the future, METTL3 and NDUFB5 may serve as therapeutic targets to ameliorate DFU progression.

### Electronic supplementary material

Below is the link to the electronic supplementary material.

Supplementary Material 1

## Data Availability

The data that support the findings of this study are available from the corresponding. author upon reasonable request.

## Abbreviations

DFU: Diabetic foot ulcer
DM: Diabetes mellitus
NDUFB5: NADH: ubiquinone oxidoreductase subunit B5
HUVECs: Human umbilical vein endothelial cells
METTL3: Methyltransferase-like 3
IGF2BP2: Insulin like growth factor 2 mRNA binding protein 2
AGEs: Advanced glycation end products
m6A: N6-methyladenosine
CCK: Cell counting kit
OCR: Oxygen consumption rate
MMP: Mitochondrial membrane potential
ROS: Reactive oxygen species
qRT-PCR: Quantitative real time PCR
WB: Western blot
RIP: RNA immunoprecipitation
STZ: Streptozotocin
HE: Hematoxylin and eosin
DUSS: Diabetic ulcer severity score
SD: Standard deviation
