# Supplementary material for: METTL3-mediated NDUFB5 m6A modification promotes cell migration and mitochondrial respiration to promote the wound healing of diabetic foot ulcer
Source: J Transl Med. 2024 Jul 9;22:643. doi: 10.1186/s12967-024-05463-6 (PMC11234709; doi:10.1186/s12967-024-05463-6)
Supplement: Supplementary file 1 — Supplementary Material 1 [file 12967_2024_5463_MOESM1_ESM.docx]

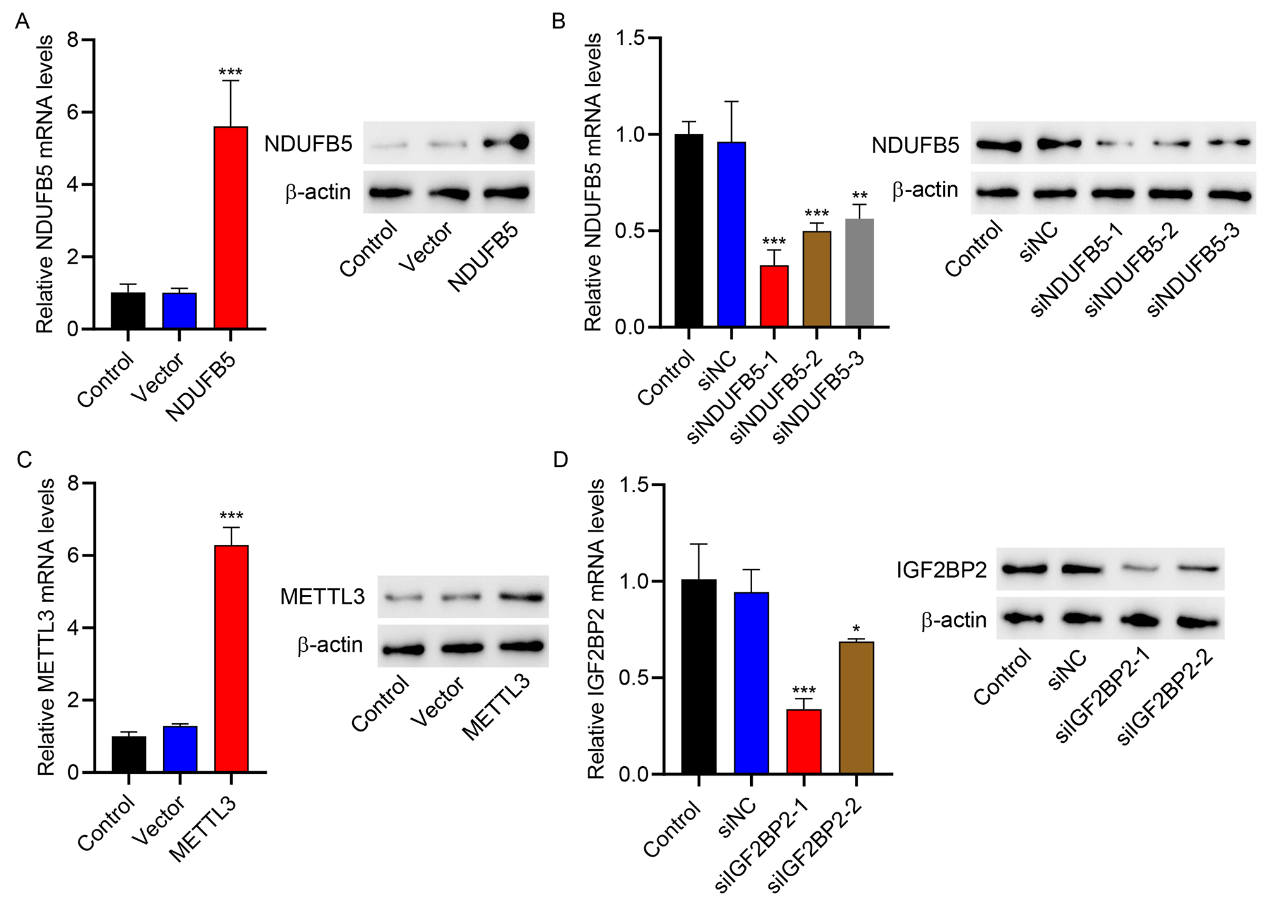


**Figure S1. Overexpression and knockdown in** **HUVECs.** (A) qRT-PCR (left) and WB (right) analysis of NDUFB5 level in HUVECs transfected with NDUFB5 expression vector. (B) qRT-PCR (left) and WB (right) analysis of NDUFB5 level in HUVECs transfected with siNDUFB5. (C) qRT-PCR (left) and WB (right) analysis of METTL3 expression in HUVECs transfected with METTL3 expression vector. (D) qRT-PCR (left) and WB (right) analysis of IGF2BP2 expression in HUVECs transfected with siIGF2BP2. ^*^*P* < 0.05, ^**^*P* < 0.01, ^***^*P* < 0.001 vs. vector or siNC group.
